# Food manufacturing foreign divestment and domestic investment in developed countries

**DOI:** 10.1016/j.heliyon.2023.e15642

**Published:** 2023-04-22

**Authors:** Justice Gameli Djokoto

**Affiliations:** Agribusiness Management Department, Central University, Ghana P.O. Box DS 2310, Dansoman, Accra, Ghana

**Keywords:** Crowding-out, Developed countries, Domestic investment, Food manufacturing, Foreign divestment, Substitution effect

## Abstract

Whilst there is some literature on the effect of inward foreign direct investment on domestic investment for the whole economy and the agricultural sector, that of foreign divestment on domestic investment for food manufacturing is rare. This paper contributes to the literature by estimating the crowding effect of foreign divestment on domestic investment in the food manufacturing sector using an unbalanced panel of 29 countries from 1991 to 2019. Foreign divestment crowded out domestic investment for developed countries in the short and long runs. In terms of the absolute reduction in domestic investment, the short-run effect is higher than the long-run effect. Policies to attract inward foreign direct investment and retain it should be pursued.

## 1 Introduction

Food manufacturing involves the conversion of relatively bulky, perishable, and typically inedible raw materials into more useful, shelf-stable, and palatable foods or potable beverages with the aid of various unit operations and technologies [1,2]. Not only does food manufacturing improve food quality and safety, but it also contributes to food security by minimising waste and loss in the food chain and by increasing food availability and marketability [[1], [2], [3], [4]]. Whilst the sector constitutes 2.10% of the gross domestic product (GDP) for developed countries, the global GDP of food manufacturing for 2017 is estimated at US$1.68 trillion [5].

Many countries pursue policies to attract foreign direct investment (IFDI) into the food manufacturing sector for some benefits including investment accumulation, technology transfer and job creation [[6], [7], [8], [9]]. However, there is evidence of foreign divestment (FD) of IFDI [[10], [11], [12], [13]]. As a strategic decision of foreign firms in a host country, FD creates changes in the business portfolio, culminating in a reduction in the level of assets [10,[14], [15], [16], [17]]. Motivated by the weak financial performance of a foreign subsidiary and other complex reasons, FD could be downsizing, relocating, and terminating [[17], [18], [19], [20]]. The partial sale or disposal of physical and organisational assets and the reduction of the workforce of the organisation is downsizing [17,21]. Relocation involves the complete shutdown of facilities and the moving of these facilities and the foreign firms' operations to another country [15,17,22]. Termination involves the complete sale or disposal of physical and organisational assets, closure of facilities, and foreign firms’ activities in a country without relocating to another country [17,23]. All these involve the reduction or complete repatriation of resources from the host country. Foreign divestment may thus, result in a reduction in investment accumulation, give rise to re-technology transfer and possible job losses. As the FD occurs in a host economy, the possible effect on domestic investment (DI) in food manufacturing is plausible.

The food manufacturing sector sources its main raw material from agriculture, a sector whose FDI has varying effects on DI; complementary [7,24], substitution [[25], [26], [27], [28]] and neutral [29,30]. Foreign divestment should also have at least one of these effects on DI in food manufacturing. Where there is no subsequent acquisition of the foreign-divested enterprise, this could lead to a crowding-out effect. On the other hand, a subsequent acquisition of the divested affiliates by affiliates of other multinational enterprises (MNEs) in the host country (existing or new entrants) or by indigenous firms and expansion of the acquisition, could result in a complementary effect of FD on DI. It may well be the case that the FD could rise just as DI such that the effect would be neutral. Considering the foregoing, what is the evidence in the case of food manufacturing in developed countries?

Afful and Kamasa [31], Baltar et al. [32], Panda and Nanda [33] and Tawiri [34] investigated the determinants of private domestic investment in manufacturing for Ghana, Brazil, Iran, India, and Libya, respectively. The determinants of DI were studied for the Group of Seven (G7) countries [[35], [36], [37]]. Whilst Boateng et al. [38] and Farla et al. [7] undertook similar investigations for Africa and developing countries, respectively, Nguyen [39] covered both developing and developed countries in separate estimations. Specifically for food manufacturing, Djokoto [40] studied the effect of FDI on DI. Some gaps are apparent in these studies. Only one study addressed the effects of FDI on DI in food manufacturing. Whilst this did not address FD, the study ignored other important control variables of DI such as trade openness, inflation, and savings rate. Further, the simultaneous role of outward foreign direct investment (OFDI) with IFDI was not accounted for. This paper examines the effect of food manufacturing FD on food manufacturing DI and accounted for OFDI, inflation, food manufacturing growth, savings rate, and trade openness. Further, the monetary value of the effect of FD on DI was computed.

The importance of domestic and foreign investment to any economy is not unknown [[6], [7], [8], [9],^41^]. Thus, policymakers have sought to promote these. However, FD which is the immediate result of a strategic decision by executives of MNEs leads to a decline in the stock of IFDI. The effect of these decisions and consequently of FD on DI has implications for the entire economy, hence, should be of interest to policymakers and economic managers.

The literature on foreign divestment is dominated by firm-level studies [18,[42], [43], [44], [45], [46], [47], [48]]. Two of the three macro-level studies examined the determinants of FD [49,50]. The third investigated the effects of FD on economic development [51]. This study focuses on the macro economy level analysis of FD on DI in developed countries for some reasons. First, developed countries initiated foreign investment [52]. Secondly, developed countries tend to possess more multinationals that engage in cross-border investments than developing and transition economies. Third, as technology and financial resources drive cross-border investments, developed countries tend to abound in these. Fourth, as countries engage in cross-border investments, these investments become candidates for foreign divestment. Consequently, we depart from the existing literature as follows: We conduct a macro-level study of the role of FD on DI. We recognised the presence of outward foreign direct investment together with inward foreign direct investment and assessed both the short and long-run effects of FD on DI. Arguably, this is the first study to address the effect of divestment on domestic investment in food manufacturing.

The results showed that FD crowded out DI for developed countries in the short and long runs. In terms of absolute reduction in DI, the short-run experienced a greater reduction than the long-run. The FD thus, appear to be a trigger for a reduction in DI. Therefore, FDI regulatory agencies should be responsive to the concerns of food manufacturing foreign investment firms. Also, where FD becomes inevitable, the agencies should require MNEs in the host country to provide advance notice to the agency on their divestment decisions. Aside from another opportunity to assist the MNEs, the notice could be circulated by the agency so that domestic or foreign firms could acquire or take over these firms. Further, the macroeconomy should be improved as the factors that promote FD could discourage IFDI. Finally, policymakers and economic managers in developed countries must target to increase domestic investment in food manufacturing by at least US1.7b annually to make up for the loss due to FD.

Next, we present theories that explain the key concepts of the paper; domestic investment and foreign divestment as well as the link between DI and IFDI. This is supported by empirical evidence. The data and sources are outlined next. The modelling together with the description and justification of the variables as well as the estimation strategy constitutes section 3. Subsequently, the results of the analysis are presented in discussed in section 4. The final section, 5, concludes the paper with some policy recommendations.

## 2 Literature review

### 2.1 Theoretical review

The theoretical review touches on the theory of domestic (aggregate) investment, FD and the effect of FDI on DI. The early literature on FD had a managerial perspective and focused on how divestment decisions are made, and what factors instigate this [11,52,53]. Porter [54] and Wilson [55] analyzed domestic barriers to exit within the industrial organisation perspective whilst the decline phase of the international product life cycle was advanced in the works of Vernon [[56], [57], [58]]. Having acknowledged the three conditions for FDI from Dunning [59] as advantages, ownership, internalisation, and location, Boddewyn [11] flipped these to formally propose a proto-theory of FD. That is, FD would occur if 1. a firm no longer holds net worthwhile advantages over firms of other nationalities; 2. it ceases to find it beneficial to use the advantages but sell or lease them to foreign firms and 3. the utilisation of its internalised net worthwhile advantages outside its home country ceases to be profitable. It is worthy of note that, whilst all the three conditions of Dunning [59] must be contemporaneously satisfied for FDI to take place, in the case of FD, at least one of the three must be satisfied. Thus, conditions for FD appear to be more easily met than that for FDI.

The many theories of investment identified in the literature include accelerator theory [[60], [61], [62], [63], [64]], Q theory [65,66] and neoclassical theory [67,68]. Agosin and Machado [29]Ahmad et al. [69], Chen et al. [70], Gallova [30], Pilbeam and Oboleviciute [71] and Wang [72] have employed the neoclassical theory in studies on the crowding effects of FDI on DI. Moreover, adjustment cost models within the neoclassical theory have gained currency and have become the workhorse of mainstream investment theory [73]. Thus, this paper focuses on the neoclassical theory. This theory acknowledges the importance of the costs of capital and output [67,68,73]. Generally, the relationship between costs of capital (interest rate) and investment is negative. This is necessary for establishing a stable full-employment general equilibrium [73]. Variants of the neoclassical theory emphasise adjustment between current and optimal capital stock. The adjustment has associated costs. These could be convex, increasing at an increasing rate in DI or non-convex [[74], [75], [76]].

Barrios et al. [77] and Wang [72] theorised that the entry of foreign firms into a host country could increase the population of domestic firms initially. The competition in the final product market would lead to price competition. This could cause domestic firms to exit the market. Where some firms produce inputs for others, the population of domestic firms would rise. In line with this theory, FD could either decrease the population of domestic firms or increase it. The resultant effect is an increase or decrease in DI. This outcome is subject to empirical examination.

### 2.2 Empirical review

The empirical review focuses on the relationship between total manufacturing private investment and total DI on one hand, and inward foreign direct investment, inflation, and growth on the other hand. Afful and Kamasa [32], Baltar et al. [32], Ebghaei [78] and Panda and Nanda [33] studied manufacturing private investments in Ghana, Brazil, Iran, and India. All except Panda and Nanda [33] applied Autoregressive distributed lag (ARDL) models whilst Panda and Nanda [33] used the general method of moments (GMM) estimator.

Afful and Kamasa [31] reported a significant negative relationship between inflation and investment in Ghana. However, Nguyen [39] found a significant positive relationship in the case of developed countries and no significant effect of inflation on investment in developing countries. The former explained that increasing prices reflect a fall in the value of the currency, hence declined desire to save in the banks resulting in a fall in investment. Exchange rate was found to be negatively related to DI in the US but neutral for France, Germany, Japan, and UK (Bahmani-Oskooee & Baek [35]).

Regarding economic growth, Afful and Kamasa [31] and Panda and Nanda [33] concluded that growth positively influenced investment in the manufacturing sector in Ghana and India, respectively. The latter noted that investment increases during periods of boom and are likely to fall during periods of recession. Economic growth positively influences investment in – France, Germany, the UK, and the US (Bahmani-Oskooee and Baek, [35]). Boateng et al., [38]; Djokoto, [27]; Farla et al. [7], and Nguyen [39], found positive effect of economic growth on DI in developing countries. Bakari [26] and Bakari et al. [37] however, found no significant relationship between economic growth and DI in France and Germany based on an ARDL estimation. For Japan, Bahmani-Oskooee and Baek [35] also reported no significant effect. There were no attributions for these findings.

Three findings of Djokoto [40], the only study on the effect of foreign direct investment on domestic investment in the manufacturing sector, are relevant here: First, developed economies experienced a crowd-out effect of FDI on DI in the short run, whilst the others experienced no significant effect. Second, food manufacturing sectors of developed, developing and transition economies exhibited a substitution effect in the long run. Third, the growth of food manufacturing value-added positively influenced domestic investment in food manufacturing.

It would be observed from the empirical review that only one study addressed the determinants of DI for food manufacturing. However, this did not address foreign divestment. This paper fills this void.

## 3 Data and modelling

### 3.1 Data

Foreign divestment is less common and somewhat ignored compared to FDI inflow or outflow [22], [78]. Thus, there were only 149 observations on FD records on food manufacturing in 29 developed countries (Appendix) from 1991 to 2019 in FAOSTAT [13]. Proceeding with the 149 could reduce the efficiency of the estimates. Thus, data on food manufacturing inward FDI (IFDI) for developed countries was used. The 149 observations of the FD were isolated using interaction with a dummy variable. Consequently, the number of observations increased from 149 to 518 for the 29 countries from 1991 to 2019. The span of the data as well as the developed countries in the data depended solely on the availability of data. The resulting observations are large enough to produce efficient estimates. The panel was unbalanced and there was no need to supply missing values as the key variable of FD must occur and must not be computed.

### 3.2 Model and modelling

The Agosin and Machado [29] model has been popular in investigating the effect of FDI on DI [40,[79], [80], [81], [82]]. However, the model has some limitations as it was developed based on assumptions for developing countries. Further, the only control variable is the economic growth rate. An alternative approach, which accounts for both outward and inward FDI simultaneously and additional control variables that are known to explain DI, is used in this study [[83], [84], [85], [86], [87], [88]]. The alternative approach accounts for other covariates of the foreign direct investment. Therefore, equation (1) is specified.

(1) $${DI}_{it}=\alpha_{0}+\alpha_{1}{L1.DI}_{it}+\alpha_{2}{L1.DI\_FD}_{it}+\alpha_{3}{IFDI}_{it}+\alpha_{4}{IFDI\_FD}_{it}+\alpha_{5}{OFDI}_{it}+\alpha_{6}FBT{TO}_{it}+\alpha_{7}{INFLA}_{it}+\alpha_{8}{GR}_{it}+\alpha_{9}{SR}_{it}+\varphi_{it}$$

Where *i* is the number of cross-sections (countries) and *t* is the period. α_k_, where k = 1, 2 … 9, are parameters to be estimated. DI is the domestic investment, L1.*DI* is one year (period) lag of *DI*. *DI_FD* is the interaction between *DI* and *FD*. *FD* is foreign divestment = 1 and zero otherwise. At the aggregate level, the *FD* of *IFDI* is negative of *IFDI* [49,51]. It is worthy of note that, UNCTAD [89], the source of the FAOSTAT data, loans to foreign affiliates are subtracted before arriving at the reported values on a directional basis captured as FDI (outward or inward). Indeed, the definition of FD as the negative of IFDI or OFDI can be found in the United Nations literature [[90], [91], [92]]. *IFDI* is inward foreign direct investment whilst *IFDI_FD* is the interaction of FD and IFDI. *OFDI* is an outward foreign direct investment and *FBTTO* is trade openness (imports plus exports divided by food manufacturing GDP). The former is necessary as it occurs together with IFDI especially, in developed countries. Food manufacturing firms import raw materials and export finished and semi-finished goods. Trade provides resources for production and market access for food manufacturing output. These must have some influence on investment. *INFLA* is the annual growth rate of the consumer price index. Increased inflation decreases the purchasing power of consumers. This would reduce consumption and increase the stock of food manufacturers. This will in turn slow down investment in food manufacturing. *GR* is the annual growth rate of food manufacturing GDP. Increased economic output would increase income for both consumption and savings. These would increase consumption and reduce the stock of manufactured goods thus, triggering additional investment in food manufacturing. *SR* is the savings rate (savings to national GDP ratio). Theoretically, the interest rate explains investment. That is, a decrease in lending interest rates will encourage investment. Simultaneously, a decrease in interest rate on savings will decrease savings and consequently, loanable funds. Thus, interest rate mediates savings and investment. Apart from *INFLA*, *GR* and *SR*, all other variables are expressed in terms of food manufacturing GDP. *INFLA* and *SR,* obtained from the World Development Indicators of the World Bank, are for the total economy as there were no corresponding series for food manufacturing. This is not out of place as the DI of food manufacturing does exist in the economy and is affected by these indicators of the total economy. The savings rate was substituted for the interest rate as there were inconsistencies in the interest rates over the period. *DI, IFDI, OFDI,* and *FD* were extracted from FAOSTAT [13]. *FBTTO* and *GR* were calculated using data obtained from FAOSTAT [13] and UN Commodity and Trade Statistics (https://comtrade.un.org/).

From equation (1), the effects of FD on DI are computed based on the combination of coefficients in Table 1. These are computed as the Wald and tested by the chi-square test statistic. The application of the chi-square test of the Wald statistic for the long run crowding effect is as follows:

a. Failure to reject the null hypothesis that η_*LR*_ =1 implies there is no crowding effect.
b. Rejection of the null hypothesis, η_*LR*_ = 1 and η_*LR*_ >1, is evidence of a crowding-in (complementary) effect.
c. Finally, rejection of the null hypothesis, η_*LR*_ = 1 and η_*LR*_ < 1, is evidence of a crowding-out (substitution) effect.In the short run
d. Failure to reject the null hypothesis that η_*SR*_ = 1 means there is no evidence of a crowding effect.
e. If the null hypothesis, η_*SR*_ = 1 is rejected and η_*SR*_ >1 then, this is a crowding-in (complementary) effect.
f. In case the hypothesis, η_*SR*_ = 1 is rejected and η_*SR*_ <1 then, it is a situation of crowding-out (substitution) effect.

**Table 1:** Computation of effects.

Effect	Computation	Null hypotheses
Short run (η_SR_)	*α*_*3*_ + *α*_*4*_	*α*_*3*_ + *α*_*4*_*= 1*
Long run (η_LR_)	*α*_1_ + *α*_2_ + *α*_3_+ *α*_4_	*α*_1_+ *α*_2_ + *α*_3_ + *α*_4_=1

Note. The -chi-square test is applicable.

#### 3.2.1 Estimation procedure

The 29 cross-sections (countries) exceeds the mean period of 16 years. Thus, the cross-sectional characteristics outweigh the time series characteristics in the model [[93], [94], [95], [96]]. Following the data structure, panel fixed, and random effects estimations were obtained. The Hausman test [97] was used to choose between fixed effects (FE) and the random effects (RE) estimations. Endogeneity could not be ruled out from the data, therefore, endogeneity was accounted for by estimating the general method of moments (GMM). The collapse option was used in the estimation of the GMM to control for instrument proliferation.

## 4 Results and discussion

### 4.1 Background of data

The mean *DI* is 0.2213 of food manufacturing GDP coinciding with Austrian data for 2010, with a minimum of 0.1061 (Greece, 2019) and a maximum of 0.5360 (Ireland, 2019) (Table 2). The mean *IFDI* is 0.0325. The minimum is −0.4209, the same as *IFDI_FD*. *OFDI* also experienced divestment to the tune of 3.0605 of food manufacturing GDP. The mean savings rate is 24% of the national GDP with a minimum of 7.5% (Greece, 2012) and a maximum of 58.8% (Ireland, 2019). On average, food manufacturing value added grew by 3.19% against a minimum of −67% reported by Romania in 2003 and a maximum of 47.3% reported by Romania in 2008.

**Table 2:** Descriptive statistics.

Variable	Observations	Mean	Standard deviation	Minimum	Maximum
*DI*	518	0.2231	0.0453	0.1061	0.5360
*L1.DI*	464	0.2229	0.0438	0.1117	0.5343
*L1.DI_FD*	464	0.0611	0.1009	0	0.3581
*IFDI*	518	0.0323	0.1243	−0.4209	1.6953
*IFDI_FD*	518	−0.0104	0.0362	−0.4209	0
*OFDI*	518	0.0490	0.2945	−3.0652	3.2737
*FBTTO*	518	3.9299	0.6223	2.0855	6.1362
*INFLA*	518	5.6889	65.9168	0.0083	1500
*GR*	518	0.0319	0.0743	−0.6787	0.4735
*SR*	518	0.2448	0.0723	0.0752	0.5878

### 4.2 Results

Table 3 reports the estimations of fixed and random effects. Both models 1 and 2 show heteroskedasticity and the existence of serial correlation. The null hypothesis of the Hausman test is rejected. Thus, the FE model is preferred to the random-effects model. Model 1 was re-estimated using pooled OLS (POLS) with Driscoll-Kraay standard errors. The POLS corrected for serial correlation whilst the Driscoll-Kraay standard errors accounted for heteroscedasticity. The average number of observations per country (time series) is 16, well below the number of cross-sections (countries) 29. The estimates of models 1 and 2 are similar. Further, the estimates of model 3 are like those of models 1 and 2. This suggests that the estimates are robust to estimators; FE, RE and POLS.

**Table 3:** Panel linear estimations.

*VARIABLES*	(1)	(2)	(3)
	*DI*	*DI*	*DI*
*L1.DI*	0.6044*** (0.0352)	0.7581*** (0.0277)	0.7595*** (0.0533)
*L1.DI_FD*	−0.0163 (0.0123)	−0.0104 (0.0120)	−0.0104 (0.0076)
*IFDI*	0.0318*** (0.0107)	0.0333*** (0.0103)	0.0332 (0.0246)
*IFDI_FD*	0.0288 (0.0369)	0.0254 (0.0353)	0.0254 (0.0536)
*OFDI*	0.0055 (0.0037)	0.0004 (0.0039)	0.0003 (0.0040)
*FBTTO*	−0.0014 (0.0021)	−0.0011 (0.0020)	−0.0011 (0.0020)
*INFLA*	−0.0001 (0.0002)	−0.0001 (0.0002)	−0.0001 (0.0001)
*GR*	−0.0605*** (0.0192)	−0.0414** (0.0182)	−0.0412* (0.0230)
*SR*	0.2390*** (0.0460)	0.0775*** (0.0181)	0.0772*** (0.0249)
CONSTANT	0.0363** (0.0151)	0.0394*** (0.0099)	0.0392*** (0.0132)
Model diagnostics			
Observations	464	464	464
Observations per country	16	16	16
R-squared	0.4608	–	0.6550
Countries	29	29	29
F test	40.44***	–	70.94***
Wald	–	853.82***	–
Estimator	FE	RE	POLS
Hausman statistics	197.27***		–
Autocorrelation test	24.062***		–
Highest VIF	1.30	1.30	1.30
Heteroskedasticity test	6628.47***	659.64***	–

Notes: 1. Values in parenthesis are standard errors, except for model 3 which are Driscoll-Kraay standard errors. 2. ***,**,* are 1%, 5% and 10% levels of statistical significance respectively. 3. FE-Fixed effects, RE-Random effects, POLS-Pooled OLS.

To explore the robustness of the estimates of model 3 to control variables, each of the control variables, one at a time, was estimated together with the key variables. The results are in Table 4. Not only are the estimates of the key variables similar across models 3 to 9, but the estimates of the control variables in models 4–9 are also like those of model 3. This is evidence of the robustness of the estimates of the key variables to the control variables. Indeed, the presence of the control variables did not significantly influence the estimates of the key variables.

**Table 4:** Robustness of fixed effects estimation to control variables.

VARIABLES	(3)	(4)	(5)	(6)	(7)	(8)	(9)
	*DI*	*DI*	*DI*	*DI*	*DI*	*DI*	*DI*
*L1.DI*	0.7595*** (0.0533)	0.7640*** (0.0599)	0.7649*** (0.0598)	0.7637*** (0.0604)	0.7656*** (0.0585)	0.7724*** (0.0577)	0.7491*** (0.0557)
*L1.DI_FD*	−0.0104 (0.0076)	−0.0021 (0.0072)	−0.0017 (0.0070)	−0.0022 (0.0074)	−0.0028 (0.0072)	−0.0037 (0.0069)	−0.0084 (0.0085)
*IFDI*	0.0332 (0.0246)	0.0440 (0.0294)	0.0434 (0.0296)	0.0440 (0.0294)	0.0439 (0.0294)	0.0439 (0.0294)	0.0339 (0.0245)
*IFDI_FD*	0.0254 (0.0536)	−0.0155 (0.0524)	−0.0164 (0.0520)	−0.0152 (0.0525)	−0.0166 (0.0515)	−0.0084 (0.0549)	0.0169 (0.0503)
*OFDI*	0.0003 (0.0040)		0.0025 (0.0023)				
*FBTTO*	−0.0011 (0.0020)			0.0006 (0.0021)			
*INFLA*	−0.0001 (0.0001)				−0.0003*** (0.0001)		
*GR*	−0.0412* (0.0230)					−0.0353 (0.0228)	
*SR*	0.0772*** (0.0249)						0.0744*** (0.0240)
CONSTANT	0.0392*** (0.0132)	0.0500*** (0.0129)	0.0497*** (0.0129)	0.0478*** (0.0137)	0.0505*** (0.0127)	0.0494*** (0.0125)	0.0362*** (0.0123)
Model diagnostics							
Observations	464	464	464	464	464	464	464
R-squared	0.6550	0.6370	0.6373	0.6371	0.6382	0.6399	0.6509
Countries	29	29	29	29	29	29	29

Notes: 1. Values in parenthesis are Driscoll-Kraay standard errors. 2. ***,**,* are 1%, 5% and 10% levels of statistical significance respectively.

Whilst it was appropriate to employ the linear panel estimators, FE and RE, endogeneity between the explained and the explanatory variables could be present. To account for this, the GMM estimator was applied to the data (Table 5). In model 10, the number of instruments was 63, far above the number of cross-sections, 29. Thus, the collapse option was used to prevent the proliferation of the instruments in the GMM estimation. The result is model 11. The probability of the second-order serial correlation is statistically insignificant. Whilst model 10 has more statistically significant estimates than model 11, the magnitude of the estimates of models 10 and 11 are generally alike. It is worth noting that the probability of the Sargan statistics in model 10 is 0.9995 whilst that of model 11 is 1.0000. Thus, controlling for instrument proliferation did not reduce the level of probability of the Sargan statistics. As both levels of probability are greater than 0.10, this suggests that the overidentifying restrictions assumed in the estimation procedure are valid. The standard errors of model 11 are finite-sample corrections as the default two-step standard errors are biased in finite samples due to the neglected sampling error in the weighting matrix based on Windmeijer's [97] approach.

**Table 5:** General method of moments (GMM) estimations.

VARIABLES	(10)	(11)
	*DI*	*DI*
*L1.DI*	0.7555*** (0.0682)	0.5475*** (0.1466)
*L1.DI_FD*	−0.0473*** (0.0066)	−0.0512 (0.0320)
*IFDI*	0.0479 (0.0577)	0.1195 (0.0950)
*IFDI_FD*	0.0248** (0.0121)	0.0437 (0.0505)
*OFDI*	0.0070** (0.0030)	0.0186* (0.0112)
*FBTTO*	0.0041* (0.0021)	−0.0009 (0.0031)
*INFLA*	−0.0002*** (0.0000)	−0.0011 (0.0011)
*GR*	−0.0880*** (0.0222)	−0.0633 (0.0927)
*SR*	0.3538*** (0.0706)	0.2152** (0.0989)
Constant	−0.0447*** (0.0120)	0.0576 (0.0465)
Model diagnostics		
Observations	464	464
Countries	29	29
Wald	7400***	–
Instruments	63	–
Probability (AR (2))	0.2425	0.2716
Sargan test (Probability)	0.9995	1.0000

Notes: 1. ***, **, * are 1%, 5% and 10% of statistical significance respectively. 2. Values in parenthesis in model 11 are Windmeijer's [97] finite-sample correction as the default two-step standard errors is biased in finite samples due to the neglected sampling error in the weighting matrix.

Another robustness to control variables was carried out, this time, with the GMM for model 11. The results in Table 6 show that the estimates of the key variables are similar across models 11 to 17. Also, the estimates of the control variables in models 12–17 are like those of model 11. Therefore, the estimates of model 11 are robust to control variables. The probability of the second-order serial correlation test and the Sargan test is also appropriate.

**Table 6:** Robustness of GMM estimations to control variables.

VARIABLES	(11)	(12)	(13)	(14)	(15)	(16)	(17)
	*DI*	*DI*	*DI*	*DI*	*DI*	*DI*	*DI*
*L1.DI*	0.5475*** (0.1466)	0.4894** (0.2332)	0.4785*** (0.1830)	0.6168*** (0.1765)	0.3973*** (0.1296)	0.3902 (0.3189)	0.4162** (0.1921)
*L1.DI_FD*	−0.0512 (0.0320)	−0.0694* (0.0403)	−0.0426 (0.0417)	−0.0638 (0.0390)	−0.0595* (0.0346)	−0.0634 (0.0471)	−0.0514 (0.0366)
*IFDI*	0.1195 (0.0950)	0.1122 (0.0931)	−0.0022 (0.1095)	0.0771 (0.0787)	0.0389 (0.0855)	0.1084 (0.1308)	−0.0309 (0.2539)
*IFDI_FD*	0.0437 (0.0505)	0.0488 (0.0381)	0.0536 (0.0451)	0.0367 (0.0264)	0.0503 (0.0460)	0.0345 (0.0887)	0.0643 (0.0528)
*OFDI*	0.0186* (0.0112)		0.0144** (0.0065)				
*FBTTO*	−0.0009 (0.0031)			−0.0004 (0.0058)			
*INFLA*	−0.0011 (0.0011)				−0.0010 (0.0012)		
*GR*	−0.0633 (0.0927)					−0.1298 (0.2286)	
*SR*	0.2152** (0.0989)						0.5539* (0.2843)
Constant	0.0576 (0.0465)	0.1170** (0.0546)	0.1160*** (0.0416)	0.0872** (0.0405)	0.1393*** (0.0311)	0.1424* (0.0791)	−0.0044 (0.0692)
Model diagnostics							
Observations	464	464	464	464	464	464	464
Countries	29	29	29	29	29	29	29
Probability (AR (2))	0.2716	0.2216	0.2904	0.2477	0.2206	0.2606	0.2809
Sargan test (Probability)	1.0000	1.0000	1.0000	1.0000	1.0000	1.0000	1.0000

Notes: 1. ***, **, * are 1%, 5% and 10% of statistical significance respectively. 2. Values in parenthesis are Windmeijer's [97] finite-sample correction as the default two-step standard errors is biased in finite samples due to the neglected sampling error in the weighting matrix.

To aid the discussion and compare the panel linear estimations to the GMM estimates, models 3 and 11 are re-presented in Table 7. Although model 3 possesses more statistically significant variables than model 11, the estimates are generally alike in sign and magnitude. This also suggests robustness between the POLS estimates and the GMM estimates. Therefore, the results obtained from fitting equation (1) to the data are robust to estimator, control variables and violations of the assumptions of linear regression.

**Table 7:** Robustness to endogeneity (fixed effects and GMM) estimations.

VARIABLES	(3)	(11)
	*DI*	*DI*
*L1.DI*	0.7595*** (0.0533)	0.5475*** (0.1466)
*L1.DI_FD*	−0.0104 (0.0076)	−0.0512 (0.0320)
*IFDI*	0.0332 (0.0246)	0.1195 (0.0950)
*IFDI_FD*	0.0254 (0.0536)	0.0437 (0.0505)
*OFDI*	0.0003 (0.0040)	0.0186* (0.0112)
*FBTTO*	−0.0011 (0.0020)	−0.0009 (0.0031)
*INFLA*	−0.0001 (0.0001)	−0.0011 (0.0011)
*GR*	−0.0412* (0.0230)	−0.0633 (0.0927)
*SR*	0.0772*** (0.0249)	0.2152** (0.0989)
CONSTANT	0.0392*** (0.0132)	0.0576 (0.0465)
Model diagnostics		
Observations	464	464
R-squared	0.6550	–
Countries	29	29
Probability (AR (2))	–	0.2716
Sargan test (Probability)	–	1.0000

Notes: 1. Values in parenthesis in model 3 are standard errors, except for model 3 which are Driscoll-Kraay standard errors. 2. ***,**,* are 1%, 5% and 10% levels of statistical significance respectively. 3. FE-Fixed effects, RE-Random effects, POLS-Pooled OLS. 4. Values in parenthesis in model 11 are Windmeijer's [97] finite-sample correction as the default two-step standard errors are biased in finite samples due to the neglected sampling error in the weighting matrix.

### 4.3 Discussion of other variables

The coefficients of IFDI are positive and statistically significant. This suggests IFDI has no discernible effect on food manufacturing DI in the short run for developed countries. This is a departure from the findings of Djokoto [40] for developed countries. The departure may be due to the reason that the model of Djokoto [40] contained fewer control variables than this study. Indeed, the inclusion of statistically significant control variables not only draws from the variability in the dependent variable but may also reduce the statistical significance of the other covariates.

The coefficients for OFDI are positive and statistically significant for model 11. One United States dollar increase in OFDI will induce a US$0.02 increase in food manufacturing DI in developed countries. This is a small effect, though. The positive sign can be attributable to joint investments of MNEs, both at home and abroad. Some goods from foreign subsidiaries could be inputs for the home factory, hence, an increase in investment abroad would lead to an increase in home investments as well.

The coefficients of food manufacturing trade openness (FBTTO) and inflation (INFLA) are statistically insignificant. Thus, these do not have a discernible effect on DI. Whilst the statistical insignificance of the coefficient departs from Afful and Kamasa [31], the sign concurs with their finding.

The coefficients of *GR*, although negative for both models, it is statistically significant for model 3 and statistically insignificant for model 11. It was expected that an increase in *GR* should spur *DI*, however, this did not turn out to be the case. Whilst this result agrees with those of Bahmani-Oskooee and Baek [35], Bakari [36] and Bakari et al. [37] respectively for Japan, France and Germany, other studies have reported positive and significant coefficients for the growth rate on private investment [7,31,33,38,39]. It must be noted that the value added is for food manufacturing. Resources from food manufacturing are inadequate for investment in food manufacturing. Additional and perhaps more substantial savings would arise from other sectors to fund investment in food manufacturing hence, the negative and statistically insignificant sign.

The coefficients of SR are positive and statistically significant for both models. A one US dollar increase in savings would induce 21 cents increase in domestic investment in food manufacturing in developed countries. The positive sign is in line with the theory that resources from households or surplus units become investable funds for industry or deficit units. Recalling that the savings rate is for the whole economy whilst the DI is for only food manufacturing, the 21 cents suggest that about 80 cents of the US$1.00 saved are channelled into sectors other than food manufacturing. The 21 cents is about 10 times the share of food manufacturing in GDP in developed in 2017 [5].

### 4.4 Crowding effect of foreign divestment on domestic investment

Except for L*1.DI*, the coefficients of all key variables are not statistically significant. The statistical significance of *L1.DI* implies previous year's DI would influence the current year's *DI*. Aside from conforming to theoretical expectations, the positive and statistical coefficient is particularly useful for GMM estimation.

The application of the Wald in Table 1 is based on estimates in models 3 and 11 and is presented in Table 8. The Wald from both the fixed effects and GMM estimations is less than 1.00. The test of difference in the Wald suggests that these are statistically not different. As the GMM accounts for endogeneity, as is common with macroeconomic variables, we focus on the GMM effects. In the short run, the null hypothesis that the Wald is equal to 1.00 is rejected. Specifically, the Wald is less than 1.00. This implies that a US$1.00 increase in FD would increase DI by US$0.1631. This shows that as much as US$0.84 (1–0.1631) of domestic investment is lost due to an increase in FD of US$1.00. This is certainly a substitution or crowding-out effect of FD on DI, in the short run.

**Table 8:** Effects of foreign divestment on domestic investment in food manufacturing.

	Fixed effects	GMM	Difference
Effects			
Short run	0.0586 [400.94]***	0.1631 [143.45]***	−1.2408 [1.54]
Long run	0.8077 [13.95]***	0.6594 [4.19]**	0.8514 [0.7250]
The monetary cost of the effects			
Short run	–	US$3.640 b/year	–
Long run	–	US$1.675 b/year	–

Notes: 1. Values in square brackets are -chi-square test statistics. 2. ***,**,* are 1%, 5% and 10% levels of statistical significance respectively.

The null hypothesis that the Wald for the long run is equal to 1.00 is also rejected. Specifically, in the long run, a US$1.00 increase in FD would induce a US$0.66 increase in DI. This implies that US$0.34 is lost in DI owing to an increase in FD. This is also a crowding-out effect or substitution effect. Thus, in the long run, FD crowds out (substitutes) DI in food manufacturing in developed countries. The crowding-out effect is unsurprising because domestic investment includes all investments in the host economy. Thus, a reduction in investments via FD would reduce DI. As all three forms of FD, downsizing, relocation, and termination [[17], [18], [19], [20]] result in the loss of IFDI from the host economy, the substitution (crowding-out) effects can be explained.

It would be observed that the magnitude of the increase in DI due to FD is higher in the long run than in the short run. That is, the substitution effect declines as one moves from the short run to the long run. In the short run, FD comes as a shock. However, in the long run, firms that have downsized could increase investments. For firms that have relocated and/or terminated their operations, firms in the host economy could acquire some resources including space and operate similar or other businesses in food manufacturing. As in the case of Wald, the monetary values of the substitution effects have a like pattern.

Some other reasons can be adduced for the reduction in DI. First, FD creates a loss of business and investment for suppliers and off-takers of foreign-divested firms. Second, the loss of jobs and income to households would lead to low savings hence reduced investment. Third, FD could lead to reverse technology transfer. Fourth, FD could lead to a reduction in exports of food manufacturers in the case of export-oriented foreign food manufacturing firms. Fifth, the decline in exports not compensated for from other sectors could lead to increased imports with associated foreign exchange pressures and imported inflation. This can dampen wealth levels and reduce savings and hence investments. The sixth reason is that the reasons for divestment may themselves be a disincentive to firms intending to invest in the economy, both local and foreign hence, would induce a decline in DI. Thus, their accentuation would lead to the loss of DI. Finally, the seventh is that these causes could have a multiplier effect on the economy which would further drive down DI.

## 5 Conclusions and recommendations

Whilst there is some literature on the effect of inward FDI on DI for the whole economy and the agricultural sector, FD on DI for the whole economy, agriculture, and food manufacturing, is minimal. This paper contributes to the literature by estimating the crowding effect of food manufacturing FD on food manufacturing DI. Further, this paper computed the changes (reduction) in DI occasioned by the FD.

FD crowded out DI for developed countries in the short and long run. In terms of absolute reduction in DI, the short run experienced a greater reduction than the long run. The FD thus, appear to be a trigger for a reduction in DI.

These conclusions necessitate some recommended actions. First, FDI regulatory agencies should be responsive to the concerns of food manufacturing foreign investment firms. Second, where FD becomes inevitable, the agencies should require MNEs in the host country to provide advance notice to the agency on their divestment decisions. Aside from another opportunity to assist the MNEs, the notice could be circulated by the agency so that domestic or foreign firms could acquire or take over these firms. Third, the macroeconomy should be improved as the factors that promote FD could discourage IFDI. Policymakers and economic managers in developed countries must target to increase domestic investment in food manufacturing by at least US1.7b annually to make up for the loss due to FD.

Further research can consider developing countries as these tend to require more investment than developed countries and FD would be a greater disservice to them than developed countries.

## Figures and Tables

**Table:** List of developed countries in the data

Austria	Estonia	Iceland	Luxembourg	Slovenia
Belgium	Finland	Ireland	Netherlands	Spain
Croatia	France	Italy	Poland	Sweden
Cyprus	Germany	Japan	Portugal	UK
Czechia	Greece	Latvia	Romania	USA
Denmark	Hungary	Lithuania	Slovakia	
